# Prepped and ready: educating caregivers to secure firearms and medications via webinars

**DOI:** 10.1007/s44192-024-00082-5

**Published:** 2024-07-23

**Authors:** Shayla A. Sullivant, Hung-Wen Yeh, Alex Hartwig, Omar Abdelmoity, Mark Connelly

**Affiliations:** 1 Department of Developmental and Behavioral Health, Children’s Mercy Hospital, 2401 Gillham Rd, Kansas City, MO 64108 USA; 2 Texas Tech University, Lubbock, TX USA; 3 Washington University, St. Louis, MO USA

**Keywords:** Firearm, Medication, Storage, Suicide, Education

## Abstract

**Background:**

Means restriction is an approach to suicide prevention that has been shown to be effective but is underutilized in the United States. For the current study, we sought to determine if a webinar-based education intervention could impact caregiver means restriction knowledge and behaviors.

**Methods:**

Nine webinars for caregivers were offered by a children’s hospital in conjunction with community groups. Education on raising teenagers was paired with information about suicide prevention, including the importance of securing medications and firearms. Participants completed surveys prior to the presentation, immediately following and two weeks later to measure change in knowledge and storage of medications and firearms. Participants were provided a safety toolkit to secure medications and firearms.

**Results:**

Of the 327 participants who completed the baseline survey, 299 and 257 completed the second and third surveys. By the conclusion of the study, 46.6% of participants reported they had disposed of unneeded medications and 44.1% had locked up medications. Among firearm owners, use of a cable gun lock rose from 13.7% to 25.8%. In addition, 40.2% of firearm owners reported learning more about how their firearms were stored at the final survey. Most participants (88.3%) strongly agreed that the presentation provided value.

**Conclusion:**

This study shows that a webinar on safe storage appears to have some impact on behavior changes for caregivers of adolescents. A controlled study could help to clarify if the webinar format or the timing during the Covid-19 pandemic might have played a role in the degree of behavior change reported.

**Supplementary Information:**

The online version contains supplementary material available at 10.1007/s44192-024-00082-5.

## Background

Injuries are the leading cause of death for youth, with firearm related injuries now surpassing motor vehicle crashes as the leading cause of death for children in the United States [1]. Improperly secured firearms increase risk of youth suicide deaths in the home [2]. Suicide attempts by ingestion, as well as years of life lost due to unintentional overdose, also have recently increased among adolescents [3]. Suicide is now the second-leading cause of death for youth in the United States [4], impacting not only mortality but also morbidity and overall cost to society [5].

Securing items used in suicide attempts has been shown through research to have a positive impact on reducing suicide deaths [6, 7]. Unfortunately, however, this approach to suicide prevention is not universally enacted [8]. Identifying interventions that effectively lead to increased means restriction at scale could have a significant impact in preventing suicides. One modeling study predicted that if just 20% of firearm owners enacted recommended storage practices, up to 32% of firearm deaths in young people could be prevented [9].

Recommending secure storage of items used in suicide attempts (means restriction) has been studied repeatedly over the years. Prior research on means restriction interventions has focused more on hospital-based education programs [10–13] than on community-based programs [14]. These programs have focused on youth who are already identified as having risk factors for suicide. However, approximately half of youth who have been suicidal have parents who are not aware of this fact [15]. As such, children and adolescents may not always be seeking care that would result in further evaluation and management of suicide risk. Many youths who die by suicide are not in mental health treatment when they die, and the majority die on their first attempt [16]. Waiting until treatment is sought may be too late to intervene. A universal rather than targeted approach to providing education about safe storage may be more likely to reduce actual deaths, although addressing firearms can be polarizing. One such program, called "Prepped and Ready," integrates means restriction education into live community-based educational presentations geared towards caregivers who are raising teens. Preliminary data from this program have demonstrated an impact of the program on safe firearm and medication storage behaviors among parents/caregivers [17].

The current study involved the development and evaluation of a webinar format for the Prepped and Ready program. The pandemic-related moratorium on group gatherings occurred quickly, so we made the choice to pivot to the webinar format. The aims of this study were to describe the adaptation from in-person to webinar format of the Prepped and Ready program, and to review outcomes in satisfaction and behavior change for participants engaging in the webinar format.

## Methods

### Study design and setting

This was a before and after study (pre-post) given the convenience sample of who participated. The webinar presentations, entitled “Prepped and Ready: Parenting into the Teen Years,” were developed by a children’s hospital and sponsored by community organizations who hosted the webinars online in two states in the Midwest. Community organizations were sought via phone calls and emails to inquire about their interest in co-hosting a webinar. Community organizations (N = 9) who agreed included a Father’s Club (N = 1), an ADHD parent support group (N = 1), mental health centers (N = 2), schools (N = 3) and community health centers (N = 2). Each site used their own communication methods, including email, newsletters, and social media to advertise the webinar to caregivers. The webinar was described as an opportunity for parents/caregivers to learn about the changes they can make to improve safety for adolescents in our community. The content of the webinar was included in all advertising so it was clear from the outset that suicide prevention would be addressed, in addition to the importance of safe storage of medications and firearms. Adults who joined the webinar comprised the potential study sample. One English-speaking adult per household raising children in their home (up to 18 years of age) at least 50% of the time qualified to participate.

### Intervention

This study was approved by the Institutional Review Board of Children's Mercy Kansas City, IRB# 340, under the guidelines of the declaration of Helsinki. Attendees were first welcomed to the live webinar, then invited to participate in a research study. All participants provided informed consent by completing an online consent form, along with a baseline survey prior to the start of the presentation. At the conclusion of the presentation, participants received an email from the study team with a link to the post-presentation survey. Participants joined the webinar from a link they had been sent by the community organization hosting the event. The events were not recorded and lasted approximately one hour.

The webinar was led by a board-certified child and adolescent psychiatrist with expertise in suicide prevention. The content of the webinar was the same for each of the nine events. Multiple topics that pertain to raising teenagers were covered in the one-hour webinar. Various topics beyond means restriction were included purposefully, with the goal of attracting parents/caregivers who may not feel that their child was at risk for suicide. The presentation began with a focus on the importance of self-care for parents/caregivers. Additional topics included the role of impulsivity and the teen brain, technology, eating disorders, vaping and substance abuse. A large focus was placed on what steps caregivers can take to address adolescents with suicidal thoughts and how to enact safe storage measures at home. Emphasis was placed on the importance of making tangible changes that could be done immediately to reduce risk within the home. Caregivers were encouraged to dispose of medications that were no longer needed, educated on how to do so, and taught to secure bottles of medications while only having limited amounts available in weekly medication storage boxes. The relative lethality of firearms was emphasized, and caregivers were strongly encouraged to secure all firearms in the home by locking them up unloaded with ammunition locked separately. The presenter emphasized from the outset that this was not about taking firearms away, but about storing them in the safest manner possible to reduce unintended deaths. At the end of the presentation, a local law enforcement officer from the host site community demonstrated proper use of a cable gun lock on a dummy firearm and reiterated the importance of securing firearms locked and unloaded with ammunition locked separately. The officer also commented on alternative storage options for firearm owners who want to have a loaded firearm quickly available for self-defense. The officer spoke for 5–10 min, followed by a 5–15 min question and answer period with the psychiatrist.

Participants were given the opportunity at the end of the webinar to provide their name and address for receiving a safety toolkit by mail. The safety toolkit included a lockable medication storage box (Jssmst Locking Medicine Box—13.8 × 8.5 × 8.2 in.), four plastic weekly medication organizers, a resealable plastic bag containing cat litter for medication disposal, and a handout that emphasized the importance of safe storage. Firearm owners also could choose to receive a cable gun lock (Total value of toolkit: $56). The cable gun locks (Master Lock 99DSPT) were donated by a local non-profit program, “Lock it for Love.”

### Measures

Participants completed a survey prior to the presentation (T0), immediately following the presentation (T1), and at 2 weeks following the presentation date (T2, the primary study endpoint). Demographics were collected on the T0 survey. An 8-item checklist of safe storage practices was administered at T0 and T2 to evaluate any change in storage practices from baseline to the study endpoint.

Participants were also asked on the T1 survey to identify what safe storage changes they planned to accomplish (e.g., “Store firearms unloaded”) and then asked at the T2 survey what changes they had made and what barriers they perceived in enacting changes. On T1, participants were asked to provide feedback on the length of the program (e.g., “Too long”, “Too short”, “Just right”) and to respond to items about the perceived value of the program using a Likert scale ranging from strongly disagree to strongly agree. On T2 parents/caregivers also completed a checklist to identify any additional actions they completed based on the presentation (e.g., “Learned more about how firearms are stored in my home”, “Talked to a friend about what I learned”, “Talked to my kids about how I could help if they were struggling.”).

### Statistical methods

Demographics and outcome measures were all categorical and summarized by frequency and percentage. The association between the planned safe storage practices at T1 and the final behaviors at T2 were assessed by the McNemar test and the changes in medication storage practices from T0 to T2 were assessed by the Stuart-Maxwell test; results of the both tests test are presented as a chi-square test statistic.

Changes in firearm storage practices between T0 and T2 were estimated by generalized linear mixed-effects models (GLMMs), which provide unbiased estimates under the missing at random (MAR) mechanism. For each practice, GLMMs included time (baseline, final) as the fixed-effect and random participant intercepts; the odds ratio associated with time indicated the change of the practice. The GLMM parameters were estimated by adaptive Gauss–Hermite approximation method using 11 quadrature points. All analyses were conducted using the R program language and the “lmerTest” package for GLMM [18–20].

## Results

### Participants

Participation across sites ranged from 8 to 151, with an average of 63 joining for each webinar. A total of 567 joined the webinar and 410 (72.3% of those joining) completed the baseline survey (T0). We further excluded 82/410 participants (20.0%) who did not provide consent and/or were not living with minors. Of the remaining 327 participants, 299 completed the post-presentation survey (T1) and 257 completed the final survey (T2). Demographics of study participants can be found in Table 1.

**Table 1:** Demographics and safety practices for the initial and final sample

	Baseline total (*n* = 327)	Final total (*n* = 257)
*Gender*		
Female	254 (77.7)	207 (80.5)
Male	73 (22.3)	50 (19.5)
*Age groups (years)*		
≤ 44	155 (47.4)	119 (46.3)
≥ 45	172 (52.6)	138 (53.7)
*Highest level of education*		
High school graduate or < HS	21 (6.4)	16 (6.2)
Bachelor's degree or higher	306 (93.6)	241 (93.8)
*Race*		
Caucasian	306 (93.6)	240 (93.4)
Non-Caucasian/other	21 ( 6.4)	17 (6.6)
*Setting lived in*		
Urban/inner city	41 (12.5)	28 (10.9)
Suburban	175 (53.5)	138 (53.7)
Rural	111 (33.9)	91 (35.4)
*Number of minors in home (*< *18)*		
≤ 2	220 (67.4)	169 (65.8)
≥ 3	106 (32.4)	87 (33.9)
Non-response	1 (0.3)	1 (0.4)
*Age range of children in homes (years)*		
0–5	61 (18.7)	46 (17.9)
6–10	146 (44.6)	112 (43.6)
11–14	202 (61.8)	163 (63.4)
15–18	163 (49.8)	127 (49.4)
*Medication storage*		
Participants who disposed of medication	–	120 (46.7)
Participants who used weekly pillboxes	–	91 (35.4)
Participants with at least some medications locked	–	115 (44.7)
*Firearm owners*^a^	161 (49.2)	132 (51.3)
Firearm(s) locked up with cable gun lock	22 (13.7)	34 (25.8)
Firearm(s) locked up with other method	111 (68.9)	80 (60.6)
Firearm(s) unlocked	38 (23.6)	18 (13.6)
Ammunition locked up with firearm	40 (24.8)	29 (22.0)
Ammunition locked up separately from firearm	58 (36.0)	64 (48.5)
Firearm(s) loaded	13 (8.1)	14 (10.6)
Firearm(s) unloaded	117 (72.7)	111 (84.1)
Firearms locked, unloaded, ammunition locked separately (Gold standard)	34 (21.1)	37 (28.0)
Uncertain how firearms are stored in our home	2 (1.2)	2 (1.5)
Inquiries about firearm storage since presentation	–	53 (40.2)

^a^Percentages for storage practices listed under “firearm owners” reflect the percent of firearm owners who endorsed the given storage practice

### Medication storage

**Fig. 1:**
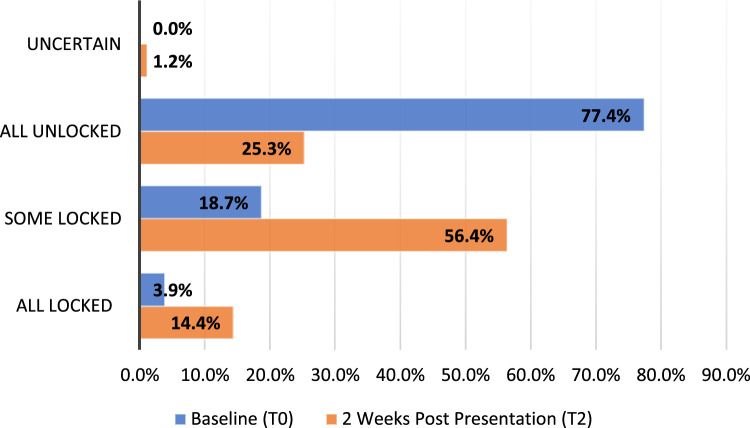
Medication storage changes

At baseline, 307/327 (93.9%) of participants reported they had at least some unlocked medication at home. Of these 307, 299 (97.4%) completed T1 and 257 (83.7%) completed T2. Of the 299 participants completing the T1 survey, 260 (87.0%) planned to dispose of medications, 239 (79.9%) planned to lock up bottles of medications, and 142 (47.5%) planned to use weekly medication storage boxes. Self-report of how medications were stored among the 247 who completed surveys at T0 and T2 are found in Fig. 1 (χ^2^ = 190.5, *p* < 0.001; Table S1).

**Fig. 2:**
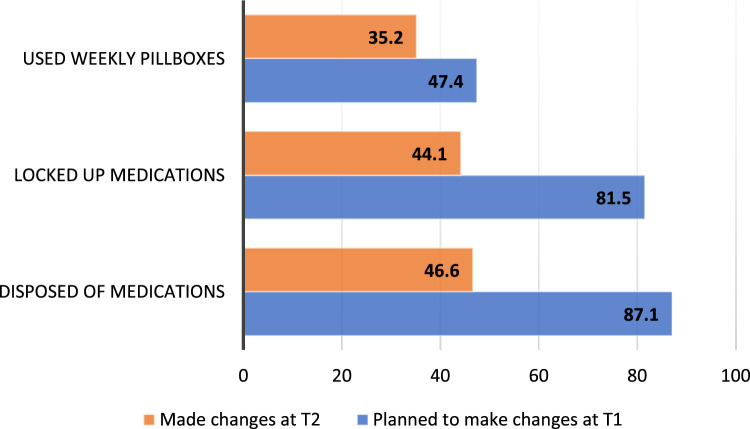
Medication storage planned change and actual change

Figure 2 shows the proportions of participants who reported completing a safe medication storage practice at T2 as a function of those who planned vs. those who had not planned to make changes at T1 (χ^2^ = 76.8, 69.1, and 8.6 for disposing, locking, and using organizers, *p* < 0.001, *p* < 0.001, and *p* = 0.003; Table S2).

Values shown for ‘made changes at T2’ indicate the percentage of participants who reported implementing the storage practice shown in the chart. Values shown for ‘planned to make changes at T1’ indicate the percentage of participants who said they planned to make changes to the listed safe medication storage practice.

### Firearm storage

At baseline (T0), 161/327 (49.2%) reported having firearms in the home, while 6/327 (1.8%) were uncertain, and 152/327 (46.5%) reported no firearms in the home. Among firearm owners at T1, 92/157 (58.6%) planned to ensure their firearms were locked up, and 65/157 (41.4%) planned to ensure their firearms were unloaded. Among the 132 firearm owners completing T2 (51.4%), 91 (68.9%) requested a cable gun lock. Among the 132 participants, 100 locked their guns at both T0 and T1, i.e. no changes. At T0, 45 (34%) stored ammunition separately from guns and 87 (68%) did not; 33 of the 87 (38%) adopted the practice at T2 while only 31 of 45 continued doing so (${\chi }_{1}^{2}=6.89, p=0.009$). Transitions of behaviors for storing guns unloaded and in the safest manner can be found in Table S4. GLMM suggested that the odds in favor of (1) the use of a cable gun lock, (2) locking ammunition separately from firearms, and (3) storing firearms unloaded increased compared to baseline (OR = 2.88, 95% CI 1.36–6.14, *p* = 0.006; OR = 2.17, 95% CI 1.18–3.96, *p* = 0.012; and OR = 2.17, 95% CI 1.14–4.12, *p* = 0.018, respectively; Table 2). The odds ratio for locking up guns in the safest manner (locked up, unloaded, and ammunition stored separately) at T2 relative to T0 was 2.02 (95% CI 0.92–4.42, *p* = 0.08).

**Table 2:** Impacts on firearm storage behaviors

Behavior	Beta	*SE*	*Z* statistic	*p* value	OR (95% CI)
Safest	0.7	0.4	1.76	0.079	2.02 (0.92, 4.42)
Using a cable gun lock	1.06	0.39	2.75	0.006	2.88 (1.36, 6.14)
Ammunition stored separately from firearms	0.77	0.31	2.51	0.012	2.17 (1.18, 3.96)
Firearms stored unloaded	0.77	0.33	2.37	0.018	2.17 (1.14, 4.12)

### Additional actions taken

At T2, 53/132 (40.2%) of participants reporting firearms in the home reported learning more about how firearms were stored at home since the presentation. In addition, on T2 surveys, 73/132 (55.3%) of firearm owners said they had talked to their kids about suicide and how they could help if they were struggling, and 89/132 (67.4%) had shared something they learned from the presentation with a friend.

### Barriers

No barriers to making changes in safe storage practices were reported by 40.9% (105/257) of the participants who completed the T2 measures, while 49.0% (126/257) reported that being busy was a barrier. Other barriers included disagreement about the storage practice by others in the family (10.1%, 26/257), not having tools to make changes happen (4.3%, 11/257), and not having a need to make changes (7.0%, 18/257).

### Satisfaction with presentation

Among participants in the webinar, 90.3% felt the length of the presentation was “just right,” while 8.4% felt it was too long and 1.3% felt it was too short. When asked if the presentation provided value, 88.3% strongly agreed, 7.4% somewhat agreed and 4% agreed. One participant somewhat disagreed that the presentation provided value. Open-ended comments provided by participants about the presentation generally supported perceived value (e.g., “My big take-away is that suicide can be prevented, and a child may only contemplate it for a brief period of time before an attempt. Locking up the meds and guns is really worth it;” “I plan to talk with my husband about this and make sure guns are put back in the safe immediately after hunting. Your mention of kids using rifles to commit suicide was new to me. I thought they generally used handguns. I liked the pictures you showed of other products to buy to lock handguns”).

## Discussion

Results of the current study on a webinar-based teen safety presentation for caregivers demonstrated that a significant fraction of caregivers reported changes in medication storage after the event, with minimal reported changes in firearm storage. Prior studies of means restriction education programs have reported variable impact. One intervention with Hispanic families in a pediatric clinic found that 61.6% removed or improved firearm storage with the education and tools provided [18]. In another study involving mostly middle-aged men participating at community events 14.8% reported changes in firearm storage, while 6.8% reported changes in medication storage [19]. The SAFETY study completed through Emergency Departments (ED) had similar outcomes to our webinars, with participants twice as likely to adopt safe storage practices with firearms and three times as likely to secure medications [12]. An ED based study found a high rate of participants using medication lockboxes (90%) after they were provided alongside education [20].

Our study results suggested that the webinar format was well-received by participants, and more than half were prompted to talk to their child or a friend about what they had learned. This study is an adaptation of our prior in-person delivery of Prepped and Ready to webinar format given the limitations of the pandemic. The interventions were not compared to one another in real time, so this must be kept in mind when comparing outcomes. The response rate of webinar participants was higher than the response rate in our prior study of conducting the Prepped and Ready program in-person (257/327 = 78.6% vs. 410/581 = 70.6% for all participants, and 132/161 = 82.0% vs. 151/220 = 68.6% among the firearm owners) [17]. The respective prevalence rates of disposing old medications, locking medications, and using medication organizers following the presentation were 45.9% vs. 56.1%, 44.5% vs. 52.7%, and 35.3% vs. 41.8%, among the webinar vs. in-person participants. The respective. odds ratios associated with baseline to final changes in favor of using cable gun locks, storing ammunition separately from firearms, storing firearms unloaded, and the safest manner were 2.9 vs. 3.6, 2.2 vs. 3.8, 2.2 vs. 3.1, and 2.0 vs. 5.9, respectively, among the webinar vs. in-person participants. The webinar participants were more likely to report the barrier of being busy than their in-person counterpart (49.0% vs. 38.0%). However, the satisfaction response was similar between the two studies [17].

The differences between the two studies could be driven by several reasons associated with selection bias. The first was the impact of the Covid-19 pandemic. In contrast to the in-person study conducted before the pandemic, the webinars were held during the pandemic, a time known to cause significant disruption in the lives of many families. It could be that caregivers were overwhelmed with managing work, school and possible illness and enacting the recommended behavior changes may simply have been more challenging during this time frame. This was reflected by busyness being a commonly endorsed barrier to enacting changes in safe storage practices. Another potential reason may be that a live in-person presentation takes more effort to attend. Participants who can leave their homes to participate in a program may have increased motivation compared to those willing to engage in a webinar from home. The former also may have more resources, including transportation and possibly childcare to attend an in-person event.

In addition, it is possible that viewers joining a webinar from home have multiple competing distractions in their environments, and thus the impact of the presentation may be less for those participants. We had no ability to gauge the engagement of the webinar viewers given they were not on camera during the presentation. We did encounter technical difficulties on occasion with the webinars, and it may have been that more participants than we realized experienced technical challenges. Finally, the safety toolkit was mailed to the participants the day following the presentation, so there was a lag between receiving the education and the tools to enact changes; this was not the case with the in-person presentations for which participant were provided toolkits to take home immediately.

Although behavior change overall with a webinar-based means restriction intervention may be lower, the possible reach may be much greater given the ability to reach some communities that are sparsely populated. For example, one site in rural Kansas hosted watch parties at five locations on the same evening. This allowed the study team to enroll 96 participants in one night, as opposed to hosting five different live presentations. The ability to engage more viewers within the same time frame, particularly in rural communities where the travel distance is limiting, is an attractive aspect of a webinar.

### Limitations

Several limitations of this project need to be considered. Our study is composed of a convenience sample of caregivers willing to participate in a webinar presentation on parenting teens and who had internet access, the majority of whom were white and educated. In addition, we were not able to control multiple factors that may have impacted outcomes. Our study design does not allow full consideration of factors that may be playing a role in our behavior change outcomes, and behavioral outcomes were based on self-report. Further, without randomizing participants to webinar versus in-person intervention format, we are unable to fully determine relative impact on behavior change outcomes of a webinar format. However, previous studies have indicated that interventions provided via online mechanisms can demonstrate robust responses [21]. Interestingly, although we were intent on reaching a more diverse sample at the outset of the current project, we found that communities in the urban core were less receptive to a webinar approach and preferred to wait for in-person event offerings. Given this, our next phase is taking an intentional approach of partnering with organizations that serve more people of color, with in-person watch parties.

## Conclusions

Even with these limitations, this study offers evidence that a webinar format of Prepped and Ready is feasible and demonstrates some efficacy, particularly with medication storage behaviors. Given the timing during a global pandemic, this study highlights how adjusting the delivery of educational information to a webinar format can be considered an alternative to reaching caregivers when an in-person presentation is not an option.

### Supplementary Information

Below is the link to the electronic supplementary material.

**Supplementary file 1.**

**Supplementary file 2.**

**Supplementary file 3.**

**Supplementary file 4.**

## Data Availability

The dataset generated and/or analyzed during the current study are not publicly available due to the sensitivity of the information in the dataset but are available from the corresponding author upon reasonable request.
