# Supplementary material for: Prepped and ready: educating caregivers to secure firearms and medications via webinars
Source: Discov Ment Health. 2024 Jul 23;4(1):25. doi: 10.1007/s44192-024-00082-5 (PMC11263524; doi:10.1007/s44192-024-00082-5)
Supplement: Supplementary file 1 [file 44192_2024_82_MOESM1_ESM.pdf]

Baseline Questionnaire

Please complete the survey below.

Thank you!

Thank you for attending the Prepped and Ready presentation.

WHAT THIS PROJECT IS ABOUT:

This project is a partnership between community organizations and Developmental and Behavioral Sciences staff at Children's Mercy Hospital. We are providing education about steps parents can take to improve the health and safety of their children as they become teens. To learn if this education is helpful, we hope to learn about your experience of attending the talk, and what you learn. We also want to understand what steps parents may take after the talk and what barriers they encounter.

WHAT YOU WILL BE ASKED TO DO:

If you agree to participate, you will complete a brief online survey before the presentation, immediately after the presentation, 2 weeks after the presentation, 1 month after the presentation, and 6 months after the presentation. An additional survey may be sent out weeks or months following the final survey. These will take approximately 5 minutes each time to complete.

ARE THERE ANY RISKS OR BENEFITS OF PARTICIPATING IN THE PROJECT?

You may not directly benefit from the project, but you may learn concepts that improve safety for youth in your home. There is some risk of discomfort to you given some of the topics (eating disorders, suicide, addiction) can be difficult. There is slight risk of loss of confidentiality when using the internet. Your confidentiality will be protected to the greatest extent possible.

WHAT WILL YOU RECEIVE FOR PARTICIPATING IN THE STUDY?

One adult from each household that participates will receive a toolkit that contains the following: four weekly medication organizers, a bag with instructions on how to dispose of unwanted medications, a pamphlet describing the importance of these steps, and a summary of the presentation with additional resources. A cable gun lock will be available to those who want one, along with education on how to use it.

PARTICIPATION IS VOLUNTARY:

Your participation in this research project is completely voluntary and you can withdraw at any time. You can stop completing the surveys at any time. There are no consequences for not completing the online surveys.

ALL ANSWERS WILL BE KEPT CONFIDENTIAL:

If you agree to participate, you will be asked to provide your e-mail address to receive a message with a link to complete the final assessment. All e-mail addresses will be stored on a secure web server and kept separate from individual responses to survey questions. An ID code will be assigned to your responses so that the survey responses remain anonymous. The project team at Children's Mercy will review the responses from the project in a summary format.

WHO I CAN CONTACT FOR INFORMATION:

If you have any questions about this project, you can contact the Research Project Coordinator, Alex Hartwig, at 816-302-3013 or ajhartwig@cmh.edu. If you have any questions about your rights as a research participant, you can contact the Children's Mercy Institutional Review Board at (816) 701-4358.

I have read the information provided. I agree to participate in this study by answering survey questions before and after the presentation. I also agree to be contacted by e-mail 2 weeks, 1 month, and 6 months from now to complete the final surveys.	◯ Yes<br>◯ No I do not want to complete the survey
Please enter your email address.	__________________________
Please enter your email address ONE MORE time for verification purposes only.	__________________________
The e-mail address you typed in DOES NOT match.<br><br>Please try again.	
Presentation was provided in a	◯ Virtual, video setting<br>◯ In-person, live setting
If you would like to receive your toolkit, you will need to provide your name and address. Once your toolkit has been sent out, your name and address will be deleted from your record.<br><br>By clicking yes, you agree to provide study team members with your name and address.	◯ Yes<br>◯ No
Name:	__________________________

Please provide the address, city, and zip code so we can mail you the toolkit.
(Disclaimer: we are unable to deliver to a P.O. Box. Please provide residential addresses only.)

Street Address: __________________________________

City: __________________________________

State: __________________________________

Zip code: __________________________________

Would you like a cable gun lock included in your toolkit?
◯ Yes
◯ No

Demographic Information

Please answer the following questions about your background.

What is your sex?

- ◯ Male
- ◯ Female
- ◯ Transgender
- ◯ Other
- ◯ Prefer not to say

What is your age?

- ◯ 18-29
- ◯ 30-44
- ◯ 45-59
- ◯ >60

Demographic Information (continued)

Highest level of education?	◯ < 12 years<br>◯ High School graduate<br>◯ College graduate or higher
What is your ethnicity?	◯ Hispanic<br>◯ Not Hispanic

Demographic Information (continued)

What is your race?

- ◯ Caucasian
- ◯ African-American
- ◯ American Indian or Alaska Native
- ◯ Asian
- ◯ Native Hawaiian or Other Pacific Islander
- ◯ Other

What is the setting where you live?

- ◯ Urban
- ◯ Inner city
- ◯ Suburban
- ◯ Rural

Demographic Information (continued)

Number of minors in your home (< 18)? __________________________________

Check the age ranges of the children you have in your home:

- ☐ 0-5
- ☐ 5-10
- ☐ 11-14
- ☐ 15-18

Safety Knowledge

This next section asks your perspectives on various topics of child safety. Please select the response that best represents your beliefs about each statement.

	Strongly Agree	Somewhat Agree	Agree	Somewhat Disagree	Strongly Disagree
Vaping, or the use of electronic cigarettes, is safer than regular cigarette smoking	○	○	○	○	○
I believe suicide is a preventable cause of death:	○	○	○	○	○
I think I would know if my child was at risk for suicide:	○	○	○	○	○

Safety Knowledge (continued)

	Strongly Agree	Somewhat Agree	Agree	Somewhat Disagree	Strongly Disagree
I believe suicide is a problem for youth in our community:	○	○	○	○	○
I think it is safe to ask young people about suicidal thoughts:	○	○	○	○	○
I believe my child could be at risk for suicide:	○	○	○	○	○

Safety Knowledge (continued)

	Strongly Agree	Somewhat Agree	Agree	Somewhat Disagree	Strongly Disagree
I feel confident I could access mental health support for my child if needed:	○	○	○	○	○
I know how to dispose of medications I no longer need:	○	○	○	○	○
I believe parents have a responsibility to create a safe home environment for adolescents in the home:	○	○	○	○	○
I feel comfortable talking about firearm storage with other adults in my home:	○	○	○	○	○

Safety Practices

This section asks about things you might do at home for safety. Please choose one answer per question that best describes what you are doing currently.

How are medications stored in your home? Note: Include over the counter and prescription medications (OTC, i.e. ibuprofen (Advil), acetaminophen (Tylenol), oxycodone (Percocet))	◯ All medication is locked up<br>◯ Some medications locked up, some unlocked<br>◯ All medication unlocked<br>◯ Uncertain
How many guns are in your home?	◯ 0<br>◯ 1 or more<br>◯ Uncertain if we have guns in the home
Are you responsible for firearm storage at your home?	◯ Yes<br>◯ No
Please check if you have any of the following in your home (Multiple options may apply):	☐ Firearm(s) locked up with cable gun lock<br>☐ Firearm(s) locked up with other method<br>☐ Firearm(s) unlocked<br>☐ Ammunition locked up with firearm<br>☐ Ammunition locked up separately from firearm<br>☐ Firearm(s) loaded<br>☐ Firearm(s) unloaded<br>☐ Uncertain how firearms are stored in our home
How are medications stored in your home? Note: Include over the counter and prescription medications (OTC, i.e. ibuprofen (Advil), acetaminophen (Tylenol), oxycodone (Percocet))	◯ All medication is locked up<br>◯ Some medications locked up, some unlocked<br>◯ All medication unlocked<br>◯ Uncertain
