# Supplementary material for: Prepped and ready: educating caregivers to secure firearms and medications via webinars
Source: Discov Ment Health. 2024 Jul 23;4(1):25. doi: 10.1007/s44192-024-00082-5 (PMC11263524; doi:10.1007/s44192-024-00082-5)
Supplement: Supplementary file 2 [file 44192_2024_82_MOESM2_ESM.pdf]

3 week follow-up company (T3)

This survey should take less than 5 minutes. We greatly value your time and feedback.

Please complete the survey below.

Thank you!

Safety Practices

This section asks about things you are doing at home for safety. Please choose one answer per question that best describes what you are doing currently.

I was sent the following after watching the videos:	☐ Toolkit (containing pill boxes, resource sheet, and Ziploc bag)<br>☐ Gun box and cable gun lock
How many guns are in your home?	◯ 0<br>◯ 1 or more<br>◯ Uncertain if we have guns in the home

Copyright © 2021. The Children's Mercy Hospital. All rights reserved. Used with Permission.

Safety Practices (continued)

	Yes	No
Firearm(s) loaded	◯	◯
Firearm(s) unloaded	◯	◯
Ammunition locked up separately from firearm	◯	◯
Firearm(s) locked up with cable gun lock	◯	◯
Firearm(s) locked up with other method	◯	◯
Firearm(s) locked up in gun box	◯	◯
Firearm(s) unlocked	◯	◯
Ammunition locked up with firearm	◯	◯
Uncertain how firearms are stored in our home	◯	◯

If using other method to store/lock up firearm, please explain: __________________________________

How are medications stored in your home? Note: Include over the counter and prescription medications (OTC, i.e. ibuprofen (Advil), acetaminophen (Tylenol), oxycodone (Percocet))

◯ All medication is locked up
◯ Some medications locked up, some unlocked
◯ All medication unlocked
◯ Uncertain

Copyright © 2021. The Children's Mercy Hospital. All rights reserved. Used with Permission.

Safety Practices (continued)

	Yes	No
Learned about firearms in our home and how they are stored	◯	◯
Removed firearms from our home	◯	◯
Used cable gun lock to secure firearm(s) in our home	◯	◯
Used other method to lock up firearm(s) in our home	◯	◯
Made sure firearms are unloaded in our home	◯	◯
Made sure ammunition is locked up in our home	◯	◯
Disposed of old medications	◯	◯
Locked up bottles of medications	◯	◯
Used pill boxes to lessen the amount of unlocked medications at home	◯	◯
Talked to my child about suicide and how I could help if they were struggling	◯	◯
I have sought out mental health treatment for my child	◯	◯
I do not have firearms in my home	◯	◯

Copyright © 2021. The Children's Mercy Hospital. All rights reserved. Used with Permission.

Safety Practices (continued)

These are the barriers that have prevented me from making changes (Check all that apply):

- ☐ Other adults in my home may disagree
- ☐ Life is busy and it's tough to get it all done
- ☐ I don't have the tools I need to make these changes
- ☐ These recommendations are unrealistic
- ☐ I don't see a need for changes
- ☐ I don't have any barriers

Copyright © 2021. The Children's Mercy Hospital. All rights reserved. Used with Permission.

Safety Knowledge

This section asks your perspectives on various topics of child safety. Please select the response that best represents your beliefs about each statement.

	Strongly Agree	Somewhat Agree	Neutral	Somewhat Disagree	Strongly Disagree
Vaping, or the use of electronic cigarettes, is safer than regular cigarette smoking:	○	○	○	○	○
I believe suicide is a preventable cause of death:	○	○	○	○	○
I think I would know if my child was at risk for suicide:	○	○	○	○	○
Going on diets is not generally recommended for youth and may cause harm	○	○	○	○	○

Copyright © 2021. The Children's Mercy Hospital. All rights reserved. Used with Permission.

Safety Knowledge (continued):

	Strongly Agree	Somewhat Agree	Neutral	Somewhat Disagree	Strongly Disagree
I believe suicide is a problem for youth in our community:	○	○	○	○	○
I think it is safe to ask young people about suicidal thoughts:	○	○	○	○	○
I believe my child could be at risk for suicide:	○	○	○	○	○

Copyright © 2021. The Children's Mercy Hospital. All rights reserved. Used with Permission.

Safety Knowledge (continued)

	Strongly Agree	Somewhat Agree	Neutral	Somewhat Disagree	Strongly Disagree
I know how to dispose of medications I no longer need:	○	○	○	○	○
I feel comfortable talking about firearm storage with other adults in my home:	○	○	○	○	○
I feel confident I could access mental health support for my child if needed:	○	○	○	○	○

Copyright © 2021. The Children's Mercy Hospital. All rights reserved. Used with Permission.

Presentation Feedback

This next section asks for your feedback about the presentation.

I learned information that is valuable to me as a parent at the Prepped and Ready presentation:

- ◯ Strongly Agree
- ◯ Somewhat Agree
- ◯ Neutral
- ◯ Somewhat Disagree
- ◯ Strongly Disagree

Any other comments you would like to share? We appreciate your feedback:

____

Copyright © 2021. The Children's Mercy Hospital. All rights reserved. Used with Permission.
