# Supplementary material for: Prepped and ready: educating caregivers to secure firearms and medications via webinars
Source: Discov Ment Health. 2024 Jul 23;4(1):25. doi: 10.1007/s44192-024-00082-5 (PMC11263524; doi:10.1007/s44192-024-00082-5)
Supplement: Supplementary file 3 [file 44192_2024_82_MOESM3_ESM.pdf]

Post Presentation Questionnaire

Thank you for completing the Baseline Questionnaire for the Prepped and Ready Research Study.

The next part in the study is to complete the Post Presentation Questionnaire below.

You will receive the 3rd questionnaire, Final Questionnaire, in two weeks.

Thank you!

Safety Knowledge

This section asks your perspectives on various topics of child safety. Please select the response that best represents your beliefs about each statement.

	Strongly Agree	Somewhat Agree	Agree	Somewhat Disagree	Strongly Disagree
Vaping, or the use of electronic cigarettes, is definitely safer than regular cigarette smoking.	◯	◯	◯	◯	◯
I believe suicide is a preventable cause of death:	◯	◯	◯	◯	◯

Safety Knowledge

	Strongly Agree	Somewhat Agree	Agree	Somewhat Disagree	Strongly Disagree
I think I would know if my child was at risk for suicide	◯	◯	◯	◯	◯
I believe suicide is a problem for youth in our community	◯	◯	◯	◯	◯
I think it is safe to ask young people about suicidal thoughts	◯	◯	◯	◯	◯

Safety Knowledge

	Strongly Agree	Somewhat Agree	Agree	Somewhat Disagree	Strongly Disagree
I believe my child could be at risk for suicide:	◯	◯	◯	◯	◯
I feel confident I could access mental health support for my child if needed:	◯	◯	◯	◯	◯
I know how to dispose of medications I no longer need:	◯	◯	◯	◯	◯

I believe parents have a responsibility to create a safe home environment for adolescents in the home:	◯	◯	◯	◯	◯

Safety Knowledge

	Strongly Agree	Somewhat Agree	Agree	Somewhat Disagree	Strongly Disagree
I feel comfortable talking about firearm storage with other adults in my home:	◯	◯	◯	◯	◯
I learned information that is valuable to me as a parent at the Prepped and Ready presentation:	◯	◯	◯	◯	◯

Planning for Change

Please answer the following questions about the changes you may make after viewing the presentation.

I plan to make changes based on what I learned from this presentation:

◯ Strongly Agree
◯ Somewhat Agree
◯ Agree
◯ Somewhat Disagree
◯ Strongly Disagree

I commit to making the following changes to make my home and community safer (Select all that apply):

☐ Inquire about firearms in my home (if uncertain)
☐ Store firearms outside my home
☐ Lock up firearms within my home
☐ Store firearms unloaded
☐ I do not have firearms in my home
☐ Dispose of unnecessary medication
☐ Lock up bottles of medication
☐ Use a pill box for daily medication
☐ Talk to my child about suicide
☐ Share something I learned tonight with a friend
☐ I do not plan to make any changes

Planning for Change (continued)

Barriers I anticipate in making these changes:

☐ Other adults in my home may disagree
☐ Life is busy and it's tough to get it all done
☐ I don't have the tools I need to make these changes
☐ These recommendations are unrealistic
☐ I don't see a need for changes
☐ I don't have any barriers

Life is busy, and choosing a time frame can help us stay true to our intentions. I plan to make these changes:

◯ Tonight
◯ Within one week
◯ Within two weeks

Presentation Feedback

This next section asks for your feedback about the presentation.

I would prefer to learn this information:

- ◯ Live webinar presentation
- ◯ Video On Demand (without live Q & A)
- ◯ Live in person presentation
- ◯ Through a newsletter

The length of this presentation was:

- ◯ Too long
- ◯ Too short
- ◯ Just right

Presentation Feedback (continued)

Any other comments you would like to share? We appreciate your feedback:

____
