# Supplementary material for: Prepped and ready: educating caregivers to secure firearms and medications via webinars
Source: Discov Ment Health. 2024 Jul 23;4(1):25. doi: 10.1007/s44192-024-00082-5 (PMC11263524; doi:10.1007/s44192-024-00082-5)
Supplement: Supplementary file 4 [file 44192_2024_82_MOESM4_ESM.docx]

Supplementary Material

**Medication Storage**

Transition of safety practices is given in Table S1, and the associations between the planned vs. the implemented behaviors are given in Table S2.

**Table S1**. Transitions of medication storage behavior

|  |  | At T2 | | | |
| --- | --- | --- | --- | --- | --- |
| At T0 |  | All unlocked | Some locked | All locked | Sub total |
|  | All unlocked | 8 | 2 | 0 | 10 |
|  | Some locked | 13 | 34 | 0 | 47 |
|  | All locked | 16 | 109 | 65 | 190 |
|  | Sub total | 37 | 145 | 65 | 247 |

**Table S2**. Association of planned vs. implemented mediation storage behaviors

| Disposing old medication | | Implemented at T2 | |  |
| --- | --- | --- | --- | --- |
|  |  | No | Yes | Sub total |
| Planned at T1 | No | 20 | 13 | 33 |
|  | Yes | 112 | 104 | 216 |
|  | Sub total | 132 | 117 | 249 |
| Locking bottles | | Implemented at T2 | |  |
|  |  | No | Yes | Sub total |
| Planned at T1 | No | 39 | 10 | 49 |
|  | Yes | 97 | 103 | 200 |
|  | Sub total | 136 | 113 | 249 |
| Disposing old medication | | Implemented at T2 | |  |
|  |  | No | Yes | Sub total |
| Planned at T1 | No | 105 | 26 | 131 |
|  | Yes | 53 | 65 | 118 |
|  | Sub total | 132 | 91 | 249 |

**Firearm Storage**

The changes in safety practice of firearm storage under different missing value mechanisms are given in Table S4.

**Table S4**. Transition of firearm storage behaviors

| Firearm locked ($\chi_{1}^{2}$ not available) | | T2 | |  |
| --- | --- | --- | --- | --- |
|  |  | No | Yes | Sub total |
| T0 | No | 32 | 0 | 32 |
|  | Yes | 0 | 100 | 100 |
|  | Sub total | 32 | 100 | 132 |
|  | |  | |  |
| Ammunition stored separately from firearms ($\chi_{1}^{2}=6.89, p=0.009$) | | T2 | |  |
|  |  | No | Yes | Sub total |
| T0 | No | 54 | 33 | 87 |
|  | Yes | 14 | 31 | 45 |
|  | Sub total | 68 | 64 | 132 |
|  | |  | |  |
| Firearm stored unloaded  ($\chi_{1}^{2}=3.69, p=0.055$) | | T2 | |  |
|  |  | No | Yes | Sub total |
| T0 | No | 8 | 26 | 34 |
|  | Yes | 13 | 85 | 98 |
|  | Sub total | 21 | 111 | 132 |
|  | |  | |  |
| Safest  ($\chi_{1}^{2}=2.37, p=0.12$) | | T2 | |  |
|  |  | No | Yes | Sub total |
| T0 | No | 86 | 18 | 104 |
|  | Yes | 9 | 19 | 28 |
|  | Sub total | 95 | 37 | 132 |
